# Anti-inflammatory and immunomodulatory effects of camel milk exosomes (CM-Exo) in rat models for burn wound healing

**DOI:** 10.22038/ijbms.2025.85157.18404

**Published:** 2025

**Authors:** Ehsaneh Azaryan, Asghar Zarban, Effat Alemzadeh, Esmat Alemzadeh, Mahdieh Rajabi-Moghaddam, Alireza Zangooie, Samira Karbasi

**Affiliations:** 1 Cellular and Molecular Research Center, Birjand University of Medical Sciences, Birjand, Iran; 2 Cardiovascular Diseases Research Center, Birjand University of Medical Sciences, Birjand, Iran; 3 Clinical Biochemistry Department, Faculty of Medicine, Birjand University of Medical Sciences, Birjand, Iran; 4 Infectious Diseases Research Center, Birjand University of Medical Sciences, Birjand, Iran; 5 Department of Biotechnology, Faculty of Medicine, Birjand University of Medical Sciences, Birjand, Iran; 6 Department of Pathology, Faculty of Medicine, Birjand University of Medical Sciences, Birjand, Iran; 7 Student Research Committee, Birjand University of Medical Sciences, Birjand, Iran

**Keywords:** Anti-oxidants, Burn, Camel milk, Exosomes, Fibroblasts, Inflammatory, Wound healing

## Abstract

**Objective(s):**

Camel milk contains proteins with several beneficial characteristics, such as immune-modulating and anti-oxidant effects. Recent research has shown that these benefits are primarily due to extracellular nanovesicles called exosomes. This study aimed to assess the wound-healing capabilities of camel milk exosomes (CM-Exo).

**Materials and Methods:**

CM-Exo was extracted, and its size and morphology were examined using DLS, TEM, and SEM. The anti-oxidant properties were assessed using a spectrophotometric (DPPH, FRAP) assay. The MTT test was used to evaluate the viability of human dermal fibroblasts (HDFs) after exposure to high concentrations (HCM-Exo) and low concentrations (LCM-Exo) of milk-Exo. Additionally, a scratch assay analyzed wound closure rate, and the expression of wound healing-associated genes (IL-6 and VEGF-A) was determined using quantitative real-time PCR. We also assessed the healing effects of a topical HCM-Exo ointment on burn-induced rat wounds over 14 days.

**Results:**

DLS, TEM, and SEM analyses showed that CM-Exo had an average size of >100 nm with a characteristic spherical shape. The average anti-oxidant activity, as measured by DPPH and FRAP assays, was higher in the HCM-Exo group compared to the LCM-Exo group. HCM-Exo and LCM-Exo enhanced the viability of HDFs, leading to quicker wound closure in an in vitro model. We found an up-regulation of essential wound healing-related genes (IL-6 and VEGF-A) indicative of an ameliorated healing effect. Evaluation of lesion size and histological data indicated a significant reduction in lesion size in the HCM-Exo and 1% silver sulfadiazine cream (Exo+SS) group compared to both the 1% silver sulfadiazine (SS) group and the negative control (Ctrl) group across days 0, 3, 7, and 14.

**Conclusion:**

Our study concluded that HCM-Exo significantly accelerated wound healing and reduced inflammatory reactions.

## Introduction

Skin tissue is the human body’s largest organ, as a barrier against diseases, regulating body temperature, and providing sensations of touch and pain (1). Burns, surgery, trauma, and diabetes complications often lead to skin tissue defects. While natural wounds heal quickly, chronic wounds impose a significant economic burden on families and communities due to ineffective treatments (2, 3). Chronic scars are often associated with deformity, limited mobility, poor appearance, or even disability, making it desirable to mitigate the complications of skin tissue defects for patients (4). Tissue repair is a complex process involving distinct but overlapping phases: homeostasis, inflammation, proliferation (granulation tissue formation), and maturation (remodeling) (5, 6). Various cells, such as macrophages, dermal fibroblasts, epidermal keratinocytes, and mesenchymal stem cells, are active in these phases. Abnormalities in each phase can prolong wound healing. For instance, scars primarily occur due to increased collagen expression and deposition (7, 8).

Researchers have recently explored various treatment methods for wound repair and regeneration. The secretion of extracellular vehicles (EVs), especially exosomes, from stem cells has been suggested as a positive factor in wound healing (9-12). EVs are lipid bilayer nanostructures that transfer cellular components like proteins, lipids, mRNA, and microRNA to receptor cells (13). Based on their intracellular origin and function, they are classified into exosomes, macrovesicles, and apoptotic bodies. Exosomes are small extracellular nanoparticles (30-150 nm) containing biological signaling molecules such as nucleic acids, proteins, and metabolites. They are released into the extracellular environment by exocytosis and mediate intercellular communication to cause physiological changes in receptor cells (13, 14). Exosomes are released by various cells, including T, B, endothelial, mast, and epithelial cells. (15). These cells are also found in physiological fluids like blood, saliva, urine, cerebrospinal fluid, and amniotic fluid (15, 16). Milk is a potential natural source for large-scale exosome production among different biofluids due to its availability, low cost, and safety (17). In the Middle East, camel milk is believed to boost the immune response and reduce cancer risk. (18). The proteins κ-casein and lactoferrin found in camel milk offer advantages such as immune support, anti-inflammatory properties, anti-oxidant effects, and antimicrobial activity (19). Recent research highlights the potential of camel milk exosomes (CM-Exo) for their anti-cancer, anti-inflammatory, anti-oxidant, and antimicrobial properties, which could be beneficial in managing various conditions and introducing new therapeutic opportunities (20).

While previous studies have explored CM-Exo’s anticancer, anti-oxidant, and anti-inflammatory properties, this study explicitly investigates their wound-healing capabilities. A topical ointment containing high concentrations of camel milk exosomes (HCM-Exo) for burn-induced rat wounds is a novel approach. Hence, this study investigates the effect of CM-Exo on burn wound healing.

## Materials and Method

Dulbecco’s modified Eagle’s medium (DMEM) and fetal bovine serum (FBS) were provided by Gibco, located in Grand Island. The penicillin and streptomycin solution and 3-(4,5-dimethylthiazol-2-yl)-2,5-diphenyltetrazolium bromide (MTT), were sourced from Sigma-Aldrich in St. Louis, MO, USA. An exosome extraction DPPH and FRAP kit were obtained from Zantox in Birjand, Iran. Merck Chemical Co supplied dimethyl sulfoxide (DMSO).

### Isolation and characterization of CM-Exo

Healthy lactating female camels in the mid-lactation period were randomly selected for this study. Milk samples were collected from five healthy camels (300 ml per animal) and stored at -80 °C until use. Exosomes were isolated from camel milk using differential centrifugation and an Exosome Isolation Kit (Zantox Kit, Iran). Initially, milk samples were centrifuged at 300 g for 30 min at 4 °C to remove fat. The supernatants were then centrifuged at 2,000 g for one hour at 4 °C, followed by centrifugation at 12,000 g for one hour, and finally at 14,000 g for two hours at 4 °C to pellet the vesicles and remove any remaining cell debris. The supernatant was filtered using 0.22-m Whatman filters (Millipore, Cork, Ireland). Subsequently, 1 ml of the filtered supernatant was mixed with 200 ml of the Zantox Exosome Isolation Kit and incubated overnight at 4 °C. After incubation, the pellet was resuspended in 200 µl of phosphate-buffered saline (PBS) following centrifugation at 12,000 g for 45 min at 4 °C. The size and morphology of the isolated exosomes were examined using Dynamic Light Scattering (DLS, Nano Brook 90Plus), transmission electron microscopy (TEM208S, Philips), and scanning electron microscopy (SEM FEI Quanta 200).

### Anti-oxidant capacity

The absolute anti-oxidant activity of high concentrations (HCM-Exo) and low concentrations (LCM-Exo) of milk-derived exosomes (milk-Exo) was evaluated using the 2,2-diphenyl-1-picrylhydrazyl (DPPH) and ferric-reducing anti-oxidant power (FRAP) assays according to the manufacturer’s instructions (Zantox kit, Iran).

### MTT assay

This study obtained human dermal fibroblasts (HDFs) from the cell bank at Iran’s Pasteur Institute (NCBI code: C646). The cells were cultured in Dulbecco’s Modified Eagle Medium (DMEM) supplemented with 10% fetal bovine serum (FBS) and 1% penicillin-streptomycin (pen/strep) at 37 °C and 5% CO_2_. The MTT assay evaluated the cytotoxic effects of HCM-Exo and LCM-Exo on HDFs. A 96-well plate was seeded with 10,000 cells per well and incubated at 37 °C with 5% CO_2_ for 24 hr. Subsequently, cells were treated with different concentrations of HCM-Exo and LCM-Exo (0, 25, 50, 100, 250, 500, and 1000 µg/ml) and further incubated for 24 hr. Afterward, 20 µl of MTT was added to each well, followed by a 4-hr incubation in darkness at 37 °C. The media was then replaced with 100 µl of dimethyl sulfoxide (DMSO; Sigma). Absorbance readings were taken at 570 and 630 nm using a microplate reader (Biotek Epoch, Winooski, VT, USA).

### Wound scratch assay

HDFs were seeded in 12-well plates at 17 × 10^4 cells per well. A scratch was introduced to each cell layer using a 200 µl pipette tip to simulate wound conditions. After removing the culture medium, the cells were washed twice with PBS to eliminate non-adherent cells and immediately treated with HCM-Exo and LCM-Exo at a concentration of 250 µg/ml. An inverted light microscope was used to evaluate the scratched areas of each well. Images were captured at 40× magnification at 0- and 24-hr post-scratch and the wound closure rate was calculated using ImageJ software.

### Real-time PCR analysis

HDFs were placed in 6-well plates at 3×10^5 ^cells per well. After reaching 70% confluence, the cells were treated with HCM-Exo and LCM-Exo at a 250 µg/ml concentration. RNA extraction was performed using the Pars Tous kit from Tehran, Iran. The quality of the RNA was evaluated using a nano drop device (BioTek Epoch). Subsequently, cDNA synthesis was carried out utilizing the Pars Tous kit. Vascular endothelial growth factor (VEGF-A) and Interleukin 6 (IL6) gene-specific primers were used in real-time analysis using the SYBR Green test (Table 1).

### Wound healing activity of HCM-Exo in vivo

***Ointment preparation***

To enhance the stability of exosomes during storage and application, the HCM-Exo ointment was formulated with a non-reactive, biocompatible base (silver sulfadiazine cream). This basis was selected due to its capacity to preserve exosome functionality and structural integrity. Before *in vivo* use, dose-response experiments on human fibroblasts were performed utilizing cell viability tests (e.g., MTT assay) to assess the potential harmful effects of high concentrations of HCM-Exo. Than the stability of the ointment was evaluated for up to 14 days at 4 °C.

The cream formulations for the three experimental groups were created in the following manner:

HCM-Exo + Silver Sulfadiazine Cream (Exo+SS) Group: Three grams of HCM-Exo was evenly mixed into 96 grams of 1% silver sulfadiazine (SS) cream base. The combination was homogenized with a sterile spatula and stirred gently to guarantee that the exosomes were uniformly distributed throughout the cream. 

Silver Sulfadiazine Cream (SS) Group: A 1% silver sulfadiazine cream was prepared as the positive control. This formulation consisted of 1 gram of silver sulfadiazine dispersed in 99 grams of a sterile, non-reactive cream base.

Control (Ctrl) Group: The negative control group received no treatment. The burn sites in this group were exposed without any cream or active substances being applied. All cream formulations were created in a sterile environment to avoid contamination and kept at 4 °C until needed. The creams› consistency and stability were checked before use. 

***Burn induction and treatments***

A total of 18 male Wistar rats, weighing approximately 150 ± 20 g, had unrestricted access to food and water in the animal house. Anesthesia was induced via intraperitoneal injection of 6 mg/kg xylazine hydrochloride (23.32 mg/ml) and 85 mg/kg ketamine hydrochloride (50 mg/ml). The dorsal hair of the rats was shaved, and the back skin was disinfected before the procedure. Skin burns were induced using the method described by Durmus *et al*. (2009) (21). Briefly, a brass probe was immersed in boiling water (100 °C) for 30 sec and then applied to the back of the rats for 30 sec without pressure. The rats were randomly assigned to three treatment groups: Ctrl group, an Exo+SS group, where burn areas were covered with Exo+SS cream once daily for 14 days; and an SS group, where burn areas were covered with batch SS cream once daily for 14 days. After 14 days of treatment, the rats were euthanized.

*The wound healing ratio*

Images of the lesions were captured on days 0, 3rd, 7th, and 14th. The wound surface area for each experimental condition was then assessed using Digimizer 4.2 software to analyze these photographs.

*Histological analysis*

At the 3^rd^, 7^th^, and 14^th^ days post-wounding, the injured area, including the epidermis, dermis, subcutaneous portions, and a small amount of adjacent intact tissue, were excised for histological examination. The skin samples were fixed in 10% neutral buffered formalin, dehydrated using gradually increasing concentrations of ethanol, cleared with xylene, and embedded in paraffin. Sections were cut at a thickness of 5 µm. The biopsies were stained with hematoxylin and eosin (H&E), and the healing process was evaluated. Images were captured using an inverted microscope (Olympus BX41, Tokyo, Japan) at 10× magnification on days 3, 7, and 14. Finally, the re-epithelialization rate was calculated using Digimizer 4.2 software.

### Statistical analyses

Statistical analysis was conducted using GraphPad Prism 9 software. Comparison between groups for normally distributed data, one-way ANOVA followed by Tukey’s post hoc test was used to compare the means of the three groups. A *P*-value of less than 0.05 was considered statistically significant. The results are presented as mean ± standard deviation (SD).

## Results

### Characterization of the isolated CM-Exo

**Figure 1:**
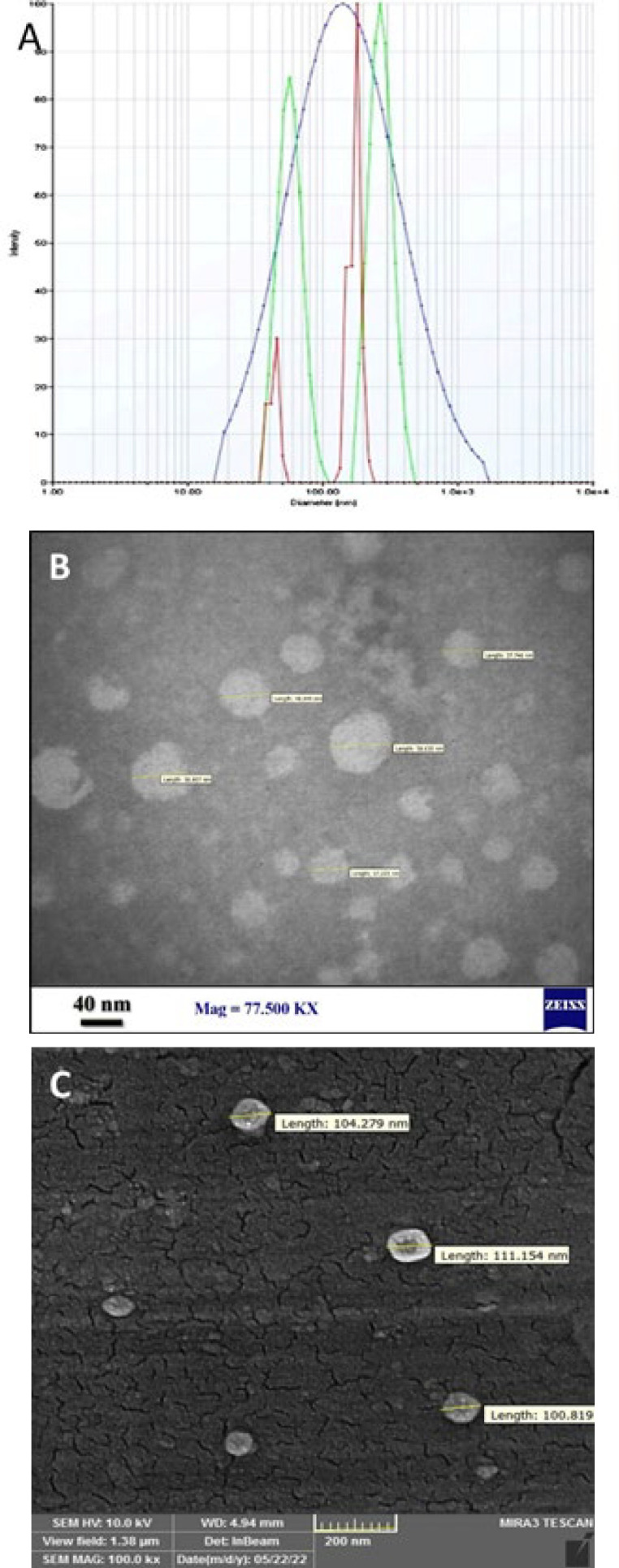
Characterization of camel milk exosome (CM-Exo)

The DLS results indicated that the average size of the exosomes was 114 ± 3.42 nm, with a polydispersity index of 0.28, revealing a relatively uniform distribution of exosome particle sizes (Figure 1A). TEM (Figure 1B) and SEM (Figure 1C) examinations revealed the presence of exosomes with average diameters of >100 nm, exhibiting a typical spherical shape.

### Anti-oxidant activity of HCM-Exo and LCM-Exo

The average anti-oxidant activity of HCM-Exo was determined to be 383.7 ± 10.6 µmol eq. Trolox/l using the DPPH assay and 261.7 ± 3.51 µmol/l using the FRAP assay. In comparison, LCM-Exo showed values of 302.0 ± 7.55 µmol eq. Trolox/l for the DPPH assay and 246.3 ± 5.68 µmol/l for the FRAP assay.

### Effect of HCM-Exo and LCM-Exo on fibroblast viability

**Figure 2:**
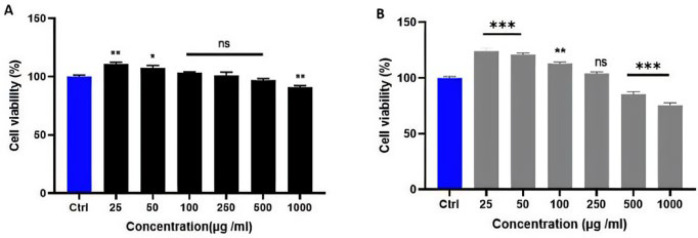
Effect of different (A) low concentrations (LCM-Exo) of camel milk-exosome and (B) high concentrations (HCM-Exo) of camel milk-exosome on cell viability in human fibroblast cells

Fibroblasts exhibited significantly higher cell viability when exposed to HCM-Exo up to 250 µg/ml compared to the control group (*P*<0.001 and *P*<0.01). However, at 250 µg/ml, there were no statistically significant differences in cell toxicity or proliferation compared to the control group (*P*>0.05). Cell viability notably decreased at higher concentrations of 500 and 1000 µg/ml (*P*<0.001). For fibroblasts treated with LCM-Exo, cell viability was significantly improved at concentrations of 25 µg/ml (*P*<0.01) and 50 µg/ml (*P*<0.05). At 100, 250, and 500 µg/ml concentrations, no significant differences in cell toxicity or proliferation were observed compared to the control group (*P*>0.05). However, at 1000 µg/ml, cell viability was substantially declined (*P*<0.01). HCM-Exo and LCM-Exo were used at a non-toxic concentration of 250 µg/ml for subsequent studies (Figure 2).

### Effect of HCM-Exo and LCM-Exo on fibroblast migration

**Figure 3:**
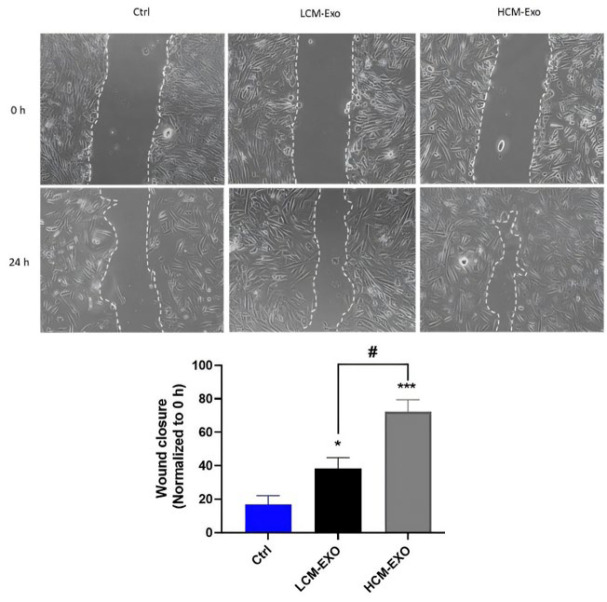
Effect of low concentrations (LCM-Exo) and high concentrations (HCM-Exo) of camel milk-exosome on cell migration in human fibroblast cells

The HCM-Exo and LCM-Exo were used to treat cells, and the change in their ability to migrate was evaluated using the wound-healing assay. The results demonstrated a significant enhancement in migration activity in the HCM-Exo-treated group compared to both the LCM-Exo-treated group (*P*<0.05) and the control group (*P*<0.001) after 24 hr of treatment (Figure 3). 

### Effect of HCM-Exo and LCM-Exo on mRNA expression of IL6 and VEGF-A

**Figure 4:**
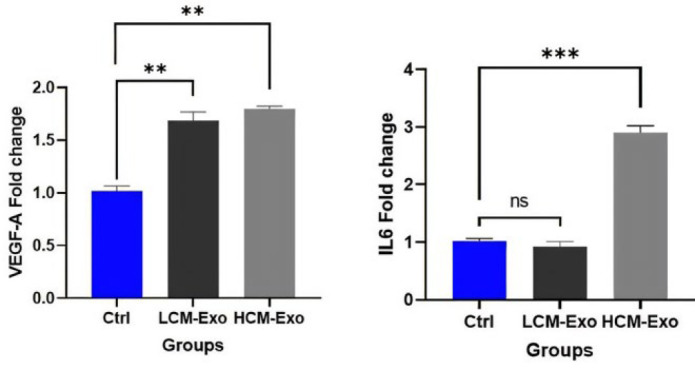
Expression of EGF-A and IL6 genes in human dermal fibroblasts (HDFs) treated low concentrations (LCM-Exo) and high concentrations (HCM-Exo) of camel milk-exosome and control group

Real-time PCR analysis (Figure 4) revealed that cells treated with HCM-Exo exhibited a significant up-regulation of IL6 (*P*<0.001) and VEGF-A (*P*<0.01) gene expression compared to the control group. In contrast, cells treated with LCM-Exo showed no statistically significant change in IL6 expression compared to the control group. However, LCM-Exo treatment resulted in a substantial increase in VEGF-A gene expression (*P*<0.01).

### In vivo studies

*Macroscopic changes in the size of the lesions*

**Figure 5:**
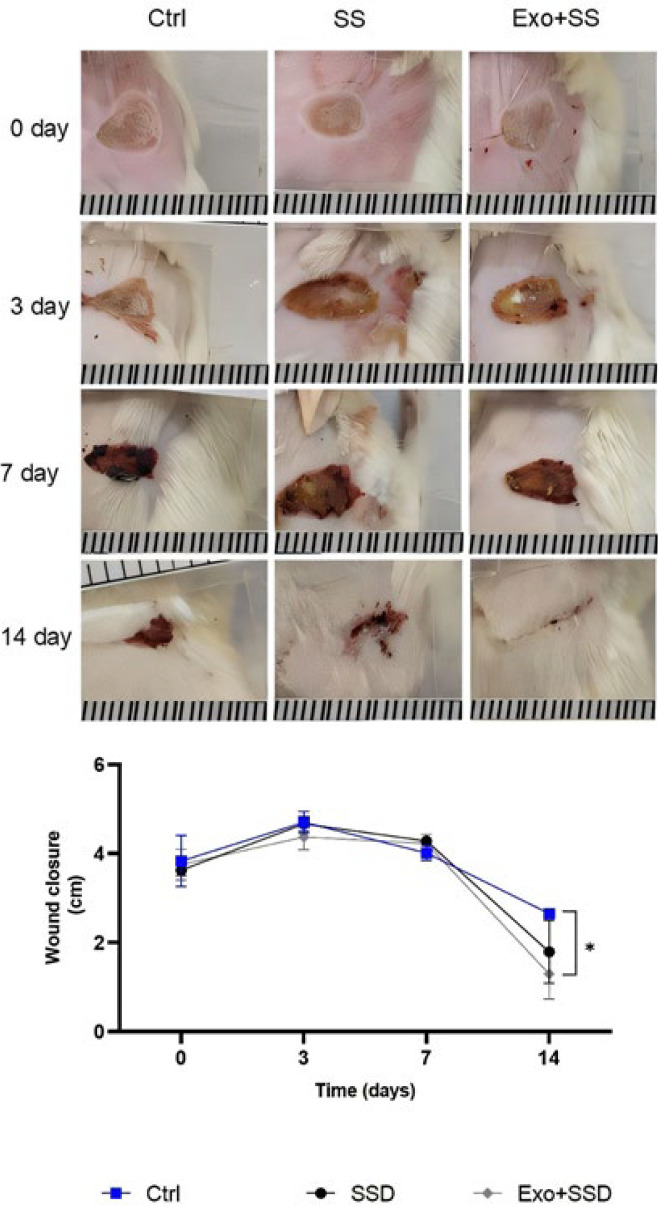
Image of wound closure rate in mouse burn wounds in the negative control (Ctrl), 1% silver sulfadiazine (SS) and exosome, and 1% silver sulfadiazine (Exo+SS) groups in 0, 3rd, 7^th^, and 14^th^ days after postwounding (mean ± SD)

The burn wounds were monitored at the 3rd, 7th, and 14^th^ days post-wounding to assess the healing status (Figure 5). Both the Exo+SS and SS groups showed a significantly more significant reduction in wound surface size compared to the Ctrl group at both 7 and 14 days after the initial wound (*P*<0.05). By the 14^th^ day post-wounding, the Exo+SS group achieved a wound surface size of 1.32 ± 0.60 cm², which was lower than both the SS group (1.80 ± 0.70 cm²) and the Ctrl group (2.30 ± 0.43 cm²).

*Histological analysis*

**Figure 6:**
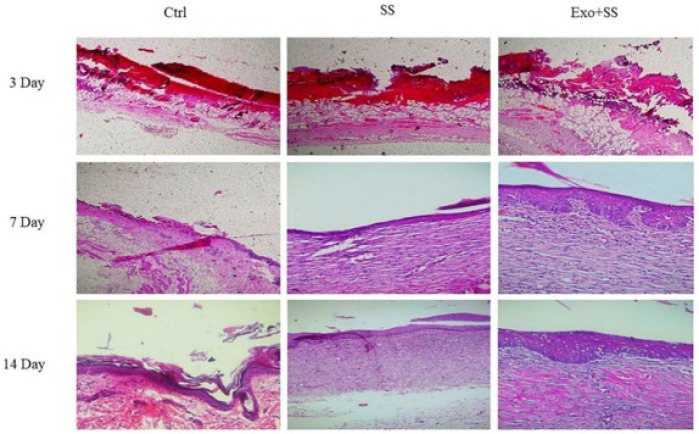
Epidermis thickness in the mouse wounds treated with negative control (Ctrl), 1% silver sulfadiazine (SS) and exosome, and 1% silver sulfadiazine (Exo+SS) groups on the 3rd, 7^th^, and 14^th ^days after the operation

Figure 6 illustrates the results of the histological examination of the wounds on the 3^rd^, 7^th^, and 14^th^ days post-wounding. On the 7^th^ day after wounding, improved re-epithelialization was observed in the group treated with high concentrations of Exo+SS compared to the other treatment groups. During this period, the Ctrl group wounds showed minimal signs of epithelialization, while the wounds in the SS group had their edges covered by a re-epithelialized epidermis. By the 14^th^ day post-injury, the wounds treated with Exo+SS exhibited the highest level of re-epithelialization, with the injured area being entirely covered by a thick epidermal layer compared to the other groups. In contrast, the SS and Ctrl groups showed incomplete epithelialization at the same time.

## Discussion

Over the past two decades, there has been a significant increase in preclinical and clinical investigations focusing on using extracellular vesicles, particularly exosomes, for skin wound healing. In line with this trend, our study aimed to isolate and characterize exosomes derived from camel milk and analyze their effects on burn wound healing. To achieve this, we used a differential centrifugation and an exosome isolation kit to isolate the exosomes. The isolated exosomes had an average diameter of>100 nm and displayed a spherical shape, consistent with the findings of Badawy *et al*. (22). However, it is noteworthy that other studies have reported varying results regarding exosome size. This variation could be attributed to differences in isolation techniques, the nature of the milk used (fresh or frozen), and the diversity of animal species considered in each study (23, 24).

In wound healing, numerous studies have explored the therapeutic potential of exosomes, particularly those derived from stem cells. However, a significant research gap exists regarding the impact of CM-Exo on burn wound treatment. Motivated by this knowledge gap, we studied exosomes’ effects on burn wounds. Our *in vitro* results indicated that HCM-Exo and LCM-Exo showed no cytotoxic effects on fibroblast cells at concentrations up to 250 and 500 µg/ml, respectively. Additionally, our study revealed that the average anti-oxidant activity, as measured by DPPH and FRAP assays, was higher in the HCM-Exo group compared to the LCM-Exo group.

Wound healing is influenced by various factors, particularly during the initial inflammatory phase, where reactive oxygen species (ROS) like HO_2_−, HO−, and O2− increase. These ROS are produced by inflammatory cells to defend against pathogens and signal the healing process. However, after their role is fulfilled, ROS levels must decrease to prevent oxidative stress, which can lead to chronic wounds. In difficult-to-heal wounds, natural anti-oxidants are often insufficient compared to healthy skin. Thus, modulating ROS through external scavenger agents may be a key therapeutic strategy to enhance wound healing (25). Due to lactoferrin and caseins, camel milk and its exosomes may offer immunomodulatory and anti-oxidant advantages. According to one study, CM-Exo and their associated genes help reduce oxidative stress, stabilize anti-oxidant levels, modulate inflammation, and enhance the immune response in rats with cyclophosphamide-induced oxidative stress and immunotoxicity (15). VEGF play a vital role in angiogenesis and support wound healing by enhancing collagen deposition and epithelialization (26). Research has shown that VEGF-A enhances blood vessel repair in diabetic animals’ ischemic limbs and significantly aids diabetic wound epithelial regeneration. Decreased VEGF-A expression impairs wound healing and vascular reconstruction (27). IL6 is vital to acute inflammation and is key to successful wound healing. It is released promptly following an injury and triggers tissue-resident macrophages, keratinocytes, endothelial cells, and stromal cells to produce proinflammatory cytokines. Additionally, IL6 is known to promote the movement of leukocytes towards a wound site (28). VEGF-A and IL6 are crucial in wound healing. IL6 boosts angiogenesis by elevating VEGF levels. (29). According to our result, HCM-Exo significantly increased up-regulation of IL6 and VEGF-A gene expression compared to the control and LCM-Exo groups. The *in vitro* results indicated that the HCM-Exo group demonstrated superior outcomes in terms of cell survival, migration, and the expression of genes associated with wound healing processes. These promising findings led us to select the HCM-Exo ointment for subsequent *in vivo *studies. During the process of skin repair, the inflammatory phase holds significant importance. The infiltration of neutrophils and monocytes/macrophages into the site of the wound primarily characterizes this phase. Our investigation into burn wound repair in rat models yielded intriguing results, revealing that exosomes influenced various immune cells during the inflammatory phase and on days 3 and 7. Notably, exosome treatment significantly reduced the number of inflammatory cells compared to the Ctrl group. These findings suggest that CM-Exo has the potential to modulate the immune response, particularly the inflammatory response, in burn wounds. As wound healing progresses from the inflammatory to the proliferative phase, different mediators are released, along with the involvement of macrophages that undergo a phenotypic transition from proinflammatory (M1) or anti-inflammatory (M2). Previous studies have demonstrated that exosomes derived from mesenchymal stem cells (MSCs) contribute to macrophage polarization through various regulatory mechanisms (30-32). These findings support the notion that CM-Exo might exhibit similar properties, potentially promoting the transition of macrophages from the M1 phenotype to the M2 phenotype. Furthermore, the administration of exosomes has been shown to suppress T-cell activation during the inflammatory phase, as reported by Su *et al*. in 2020. This highlights the immunomodulatory potential of exosomes in mitigating excessive inflammation during wound healing (33). Additionally, a study in 2016 revealed that MSC-derived exosomes expressed MiR-181C, which effectively inhibited the TLR4 signaling pathway and reduced inflammation in rats with burns (34). These findings suggest that CM-Exo may harbor similar capabilities, offering opportunities for reducing inflammation and promoting healing in burn wounds. Consistent with previous investigations, the findings of our study provide further evidence suggesting that the treatment group exhibited significantly higher angiogenic activity on day 7 compared to the control group. This indicates that exosomes play a crucial role in promoting wound closure during the proliferation phase by activating endothelial cells and fibroblasts, leading to angiogenesis and the deposition of extracellular matrix. In support of these findings, Zhang *et al*. reported that exosome treatment resulted in a notable acceleration of keratinocyte proliferation, ultimately leading to faster wound closure. This effect was attributed to the activation of the AKT/HIF-1α signaling pathway. This pathway plays a vital role in regulating cellular processes, including cell proliferation and survival, and its activation by exosomes contributes to the enhanced healing observed in our study (35). Moreover, the reconstruction of the vascular network is a critical aspect of wound healing. It has been documented that exosomes derived from MSCs can induce the production of various angiogenic factors, such as VEGF, HGF, and Ang1(36, 37). These factors play key roles in promoting the formation of new blood vessels, thereby facilitating the reestablishment of a functional vascular network within the wound site. Furthermore, a study highlighted another mechanism through which exosomes contribute to angiogenesis. Their study revealed that exosomes enhance endothelial cell proliferation by suppressing the MEF2C signaling pathway. By inhibiting MEF2C, exosomes effectively stimulate endothelial cell proliferation, facilitating the formation of new blood vessels and supporting the angiogenic process (38). Our study corroborates previous research by demonstrating that exosomes exert a significant influence on angiogenesis during the wound-healing process. Through the activation of endothelial cells fibroblasts and the modulation of various signaling pathways, exosomes promote angiogenesis, accelerate wound closure, and contribute to the reconstruction of the vascular network. These findings underscore the potential of exosomes as a promising therapeutic approach for enhancing angiogenesis in the context of wound healing.

The final crucial phase in the wound healing process is the remodeling phase, during which the extracellular matrix undergoes a series of physical and physiological changes to restore the tissue to its normal state of homeostasis. This phase typically commences around day 14 and can persist for several years. Our study results showed that the exosome treatment group exhibited a significantly higher rate of wound closure on days 3, 7, and 14 compared to the control group. A closer examination of the wounds on day 14 revealed a substantial increase in epidermal thickness in the treatment group, further validating the therapeutic effect of CM-Exo in promoting wound repair and regeneration. Exosomes exhibit tangible therapeutic benefits throughout the proliferation and remodeling phases of wound healing. They have been shown to modulate the ratio of matrix metalloproteinases and their inhibitors during the remodeling or maturation phase, thereby facilitating desirable health outcomes. Moreover, exosomes directly enhance the functionality of wound bed fibroblasts, stimulating their proliferation and migration through various mechanisms. For instance, activation of the PTEN pathway or stimulation of Notch signaling has been identified as key mechanisms through which exosomes improve the capabilities of fibroblasts involved in wound healing (39-41). These findings highlight the multifaceted role of CM-Exo in promoting wound healing during the remodeling phase. By modulating the extracellular matrix, enhancing the proliferation and migration of fibroblasts, and influencing critical signaling pathways, exosomes contribute to reorganizing and restoring tissue structure. This underscores the therapeutic potential of CM-Exo as a valuable intervention for promoting successful wound healing and achieving optimal tissue regeneration outcomes.

**Table 1:** Sequences of primers used for real-time PCR to investigation gene expression

Name	Forward	Reverse
VEGF-A	AGGGCAGAATCATCACGAAGT	AGGGTCTCGATTGGATGGCA
IL-6	AGACTTGCCTGGTGAAAATCA	GCTCTGGCTTGTTCCTCACT
GAPDH	CGAACCTCTCTGCTCCTCCTGTTCG	CATGGTGTCTGAGCGATGTGG

(A) Dynamic Light Scattering (DLS) graph, (B) , transmission electron microscopy (TEM), and (C) scanning electron microscopy (SEM) image

Significant differences compared to control were indicated: *P<0.05; **P<0.01; ***P<0.001. Ctrl, Control; ns, nonsignificant.

***P<0.001 and *P<0.05; compared to Ctrl group. ≠*P*<0.05; compared to LCM-Exo and HCM-Exo group.

**P<0.01, ***P<0.001 for each group compared to the control group.

## Conclusion

Our research findings prove that exosomes are crucial in expediting wound healing while reducing inflammatory responses. These remarkable outcomes suggest that CM-Exo holds promising potential as an effective clinical intervention for tissue reconstruction and wound healing.

## Declaration

We have not used any AI tools or technologies to prepare this manuscript.
